# Topological Structures and Membrane Nanostructures of Erythrocytes after Splenectomy in Hereditary Spherocytosis Patients via Atomic Force Microscopy

**DOI:** 10.1007/s12013-016-0755-4

**Published:** 2016-08-24

**Authors:** Ying LI, Liyuan Lu, Juan LI

**Affiliations:** 1 Department of Hematology, First Affiliated Hospital of Sun Yat-sen University, Guangzhou, 510080 Guangdong China; 2 Department of Electronic Engineering, College of Engineering, Chinese University of Hong Kong, Shatin city, Hong Kong

**Keywords:** Atomic force microscopy, Hereditary spherocytosis, Erythrocytes

## Abstract

Hereditary spherocytosis is an inherited red blood cell membrane disorder resulting from mutations of genes encoding erythrocyte membrane and cytoskeletal proteins. Few equipments can observe the structural characteristics of hereditary spherocytosis directly expect for atomic force microscopy In our study, we proved atomic force microscopy is a powerful and sensitive instrument to describe the characteristics of hereditary spherocytosis. Erythrocytes from hereditary spherocytosis patients were small spheroidal, lacking a well-organized lattice on the cell membrane, with smaller cell surface particles and had reduced valley to peak distance and average cell membrane roughness vs. those from healthy individuals. These observations indicated defects in the certain cell membrane structural proteins such as α- and β-spectrin, ankyrin, etc. Until now, splenectomy is still the most effective treatment for symptoms relief for hereditary spherocytosis. In this study, we further solved the mysteries of membrane nanostructure changes of erythrocytes before and after splenectomy in hereditary spherocytosis by atomic force microscopy. After splenectomy, the cells were larger, but still spheroidal-shaped. The membrane ultrastructure was disorganized and characterized by a reduced surface particle size and lower than normal Ra values. These observations indicated that although splenectomy can effectively relieve the symptoms of hereditary spherocytosis, it has little effect on correction of cytoskeletal membrane defects of hereditary spherocytosis. We concluded that atomic force microscopy is a powerful tool to investigate the pathophysiological mechanisms of hereditary spherocytosis and to monitor treatment efficacy in clinical practices. To the best of our knowledge, this is the first report to study hereditary spherocytosis with atomic force microscopy and offers important mechanistic insight into the underlying role of splenectomy.

## Introduction

Hereditary spherocytosis (HS) is by far the most common congenital hemolytic anemia in Northern European descendants [1]. The hallmarks of the disease are anemia, jaundice, and splenomegaly; with the age of onset of clinical symptoms, their severity are relatively variable. Severe HS patients may also have growth retardation and hemolytic crises. HS is characterized by spherical red blood cells (RBCs, or erythrocytes) in the peripheral blood rather than the normal biconcave RACs. Recent researches has discovered erythrocytes in HS are deficient in certain structural proteins including α- and β-spectrin, ankyrin, protein 4.1, and etc [2]. Such deficiencies result in cytoskeleton disruption and/or reorganization. This manifests as membrane instability that force the cells to occupy the smallest volume possible, i.e., a sphere. Therefore, the rounded erythrocytes (spherocytes) have impaired deformability and are more prone to hemolysis than normal RBCs. Moreover, spherocytes are trapped during their passage through the spleenic pulp and are selectively destroyed. Currently, there is no cure for the genetic defects underlying HS, and splenectomy and folic acid supplements are the most effective treatment for chronic symptoms of anemia and moderate to severe splenomegaly cases [3].

There are several tools to investigate the surface of RBCs. Flow cytometry measures optical and fluorescence characteristics of single cells and resolves physical properties such as size (represented by forward angle light scatter) and internal complexity (represented by right-angle scatter) of certain cell populations. Additionally, fluorescent dyes or antibodies conjugated to fluorescent dyes may bind or intercalate with different cellular components or bind specific proteins on cell membranes or inside cells [4]. For example, flow cytometry has been increasingly used for the detection and quantification of fetal red cells in maternal blood [5–8]. Antibodies to CD55 and CD59 are specific for decay-accelerating factor and membrane-inhibitor of reactive hemolysis, respectively, and can be analyzed by flow cytometry to make a definitive diagnosis of PNH [9–11]. Reticulocyte counting using fluorescent dyes that bind the residual RNA such as thiazole orange provides excellent discrimination between reticulocytes and mature RBCs with greater precision, sensitivity, and reproducibility than the traditional method [12, 13]. Light microscopy offers rapid information on morphology, but the resolution is roughly limited to half the wavelength of light. In electron microscopy, nanometer-scale resolution can easily be achieved on negatively stained samples. However, none of the three methods can make precise structural measurements along the *z*-axis, which atomic force microscopy (AFM) can facilitate.

In past several decades, AFM has become a powerful technique for studying the mechanical properties (stiffness, viscoelasticity, hardness, and adhesion) of various biological materials. The unique combination of high-resolution topography and operation under physiological environments has made it useful in many studies of cell properties [14, 15]. Though AFM has been employed to image RBCs in lab for over a decade [16, 17]. The changes of HS patients are still remained unknown.

AFM uses a sharp probe (tip) mounted to the end of a flexible cantilever that deflects when interacting with the cell surface. The cells can be scanned in *X* and *Y* directions via a piezoelectric scanner. Changes in height (*Z* direction) due to tip interactions with the cell surface are detected with a laser and a position-sensitive detector (i.e. photodetector) [14]. AFM allows quantitative measurement of the RBCs surface mechanical properties in various disease conditions[18–21] and chemical stimuli [22–24].

However, reorganization of the topological structures and membrane nanostructure of RBCs after splenectomy, especially in HS patients, has not yet been investigated and may offer critical insight into the mechanism of action. Here, we used AFM to probe RBC nanoscale surfaces and compared the morphology and membrane nanostructure of these cells before and after splenectomy. The results will facilitate a better understanding of the impact of splenectomy on RBCs from HS patients.

## Materials and Methods

### Participants

Three patients (one male and two female, aged 19, 23, and 27 years, respectively) diagnosed with HS in August 2013 at The First Affiliated Hospital, Sun Yat-Sen University were enrolled in this study. Three healthy controls (one male and two female, aged 20, 23, and 27 years, respectively) were also included. The patients were diagnosed of HS according to Guidelines for the diagnosis and management of hereditary spherocytosis—2011 update [25]. All studies were approved and conducted in accordance with Internal Review Board of the hospital. Proper informed consent forms were obtained from all blood donors in accordance with the principles of the Declaration of Helsinki on Biomedical Research. None of the patients had any complications or co-morbidities. All three patients presented with anemia, jaundice and splenomegaly. They underwent splenectomy after diagnosis with HS.

### Sample Preparation

Before and 3 months after splenectomy, 2 ml of blood was drawn from the cubital vein in the early morning—1 ml in an EDTA tube for whole blood cell counting and classification and another 1 ml in a heparin tube, which was centrifuged at ×300 g for 10 min. 2 ml of blood from healthy controls were drawn from the cubital vein in the early morning just as the patients. For the heparin tube, the supernatant was discarded (plasma, platelets, and white blood cells) and the remaining erythrocyte pellet was suspended in 0.01 M phosphate buffered saline (PBS). The cell concentration was adjusted to 1~2 × 10^11^ cells per liter with a hemocytometer. Five microliter of the RBC suspension was added to a clean, Cell-Tak adhesive treated class coverslip, fixed with 1 % glutaraldehyde for 15 min, and rinsed with deionized water 2 to 3 times. The samples were then air dried for AFM analysis.

### AFM Imaging and Measurement

Topographic images of erythrocytes were obtained from AFM (Autoprobe CP Research, Veeco) in tapping mode. A gold-coated silicon nitride tips (UL20B; Park Scientific Instruments) with a spring constant of 2.5 N/m and a tip diameter of 20 nm were used in all experiments. An optical microscope was used to help select the desired cells and direct the position of the AFM tip. The samples were placed on the AFM sample stage. The location of the cell and AFM tip were identified with light microscopy. The cells were scanned randomly in the air using tapping mode. Five cells from each sample were scanned, and the scans were repeated for a total of three times. AFM topographic images were processed and analyzed using XEI software (Park Systems Corp, Korean) and a smoothing filter was applied to remove the low frequency noise on scanning directions. The erythrocyte width (*W*) and length (*L*) represented the minimum and maximum diameter of red blood cells, respectively. The term *r* was the *L* to *W* ratio. The value of *h* and *H* represented the minimum and maximum height of the cell. The valley to peak distance (Rp-v) represented the difference between the minimal and maximal height on the *Z*-axis in the area analyzed. Ra was the average cell surface roughness.

### Statistical Analysis

The RBC morphology, cell surface topological structures and membrane nanostructure were expressed as mean ± standard deviation (SD). All the data were analyzed using SPSS 18.0 software (SPSS Inc.; Tokyo, Japan). The morphological parameters of erythrocytes collected from healthy volunteers, and HS patients before and after splenectomy were compared using a univariate analysis of variance method. A *p* value less than 0.05 was considered statistically significant.

## Results

### RBC-related Parameters from Complete Blood Count Tests in HS Patients before and after Splenectomy

**Fig. 1:**
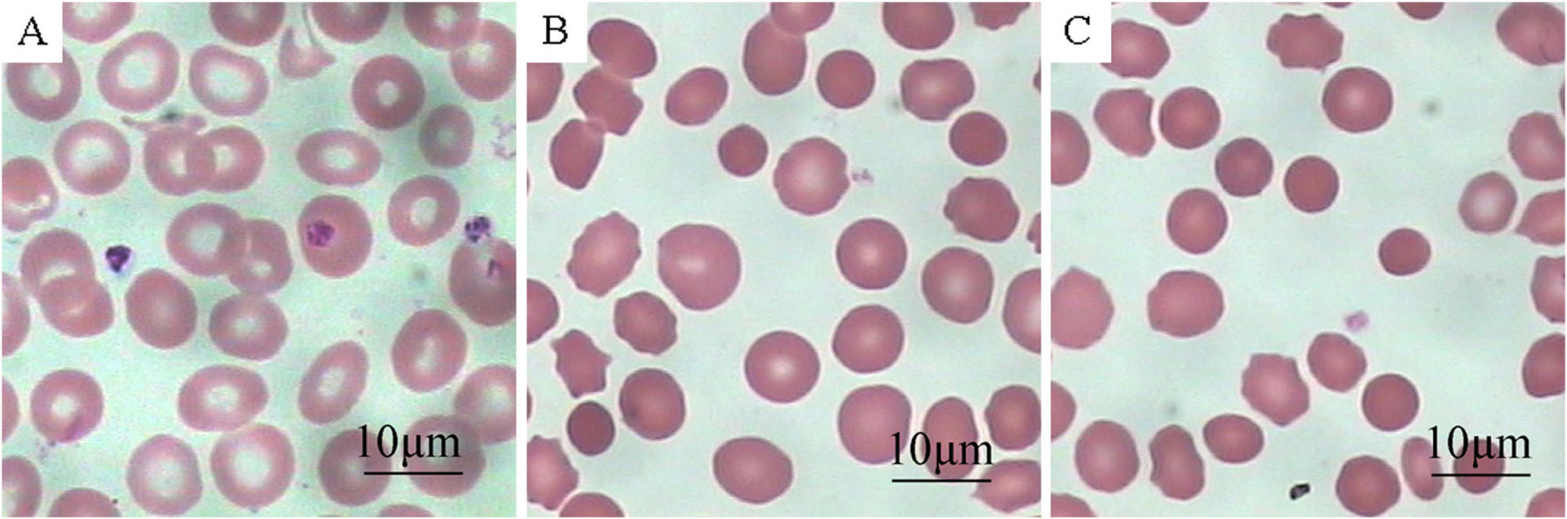
Optical microscopy images of peripheral blood smears of normal control **a** HS before splenectomy **b** and HS after splenectomy **c**. Scale bar: 10  μm (Giemsa and Wright staining, original magnification: ×1000)

All three HS patients were anemia with decreased RBC counts, hemoglobin level, and mean corpuscular volume (MCV), and increased mean corpuscular hemoglobin concentration (MCHC), percentage of reticulocyte counts, and red blood cell distribution width (RDW) (Table 1). All patients had normal serum ferritin levels, and were negative for the thalassemia gene mutation tests and were positive in the osmotic fragility tests. All three patients had family histories of HS and showed an increased number of spherocytes in the peripheral blood smear (16 %, 23 %, and 20 %; Fig. 1). After HS had been diagnosed, the patients underwent surgeries to treat HS. After surgery, the patients had increased RBC, Hb, MCV values, and decreased MCHC, percentage of reticulocyte count and RDW (Table 1).

**Table 1:** Characteristics of routine blood test of HS patients before and after splenectomy

	RBC (×10^12^)	Hb (g/L)	MCV (fL)	MCHC (g/L)	Ret (%)	RDW (%)
Pre-SE	2.45 ± 0.11**	72.00 ± 5.66**	84.55 ± 4.45*	348.00 ± 5.66**	26.67 ± 1.53**	22.00 ± 3.83**
Post-SE	4.22 ± 0.67	125.30 ± 24.90	89.47 ± 5.24	331.80 ± 31.79	1.77 ± 0.25**	17.33 ± 4.62
Control	4.36 ± 0.27	133.30 ± 9.07	91.53 ± 0.31	333.67 ± 1.15	0.67 ± 0.58	13.00 ± 1.00

*RBC* red blood cell count, *Hb* hemoglobin, *MCV* mean corpuscular volume, *MCHC* mean corpuscular hemoglobin concentration, *Ret* reticulocytes, *RDW* red blood cell distribution width

* *P* < 0.05 compared to the control group; ** *P* < 0.01 compared to the control group

Representative peripheral blood smear images from healthy controls and HS patients before splenectomy and after splenectomy were shown in Fig. 1. The HS patients before splenectomy had many morphologically abnormal and small RBCs (about 6 μm in diameter). These cells lacked a central zone of pallor and had a spheroidal shape rather than a biconcave shape (Fig. 1b). After splenectomy, the percentage of abnormal RBCs was not reduced compared to the pre-splenectomy patients. The sizes of the abnormal RBCs were variable, and the central zone of pallor were still lacking in these cells (Fig. 1c).

### AFM Morphological Parameters of RBCs in HS Patients before and after Splenectomy

**Fig. 2:**
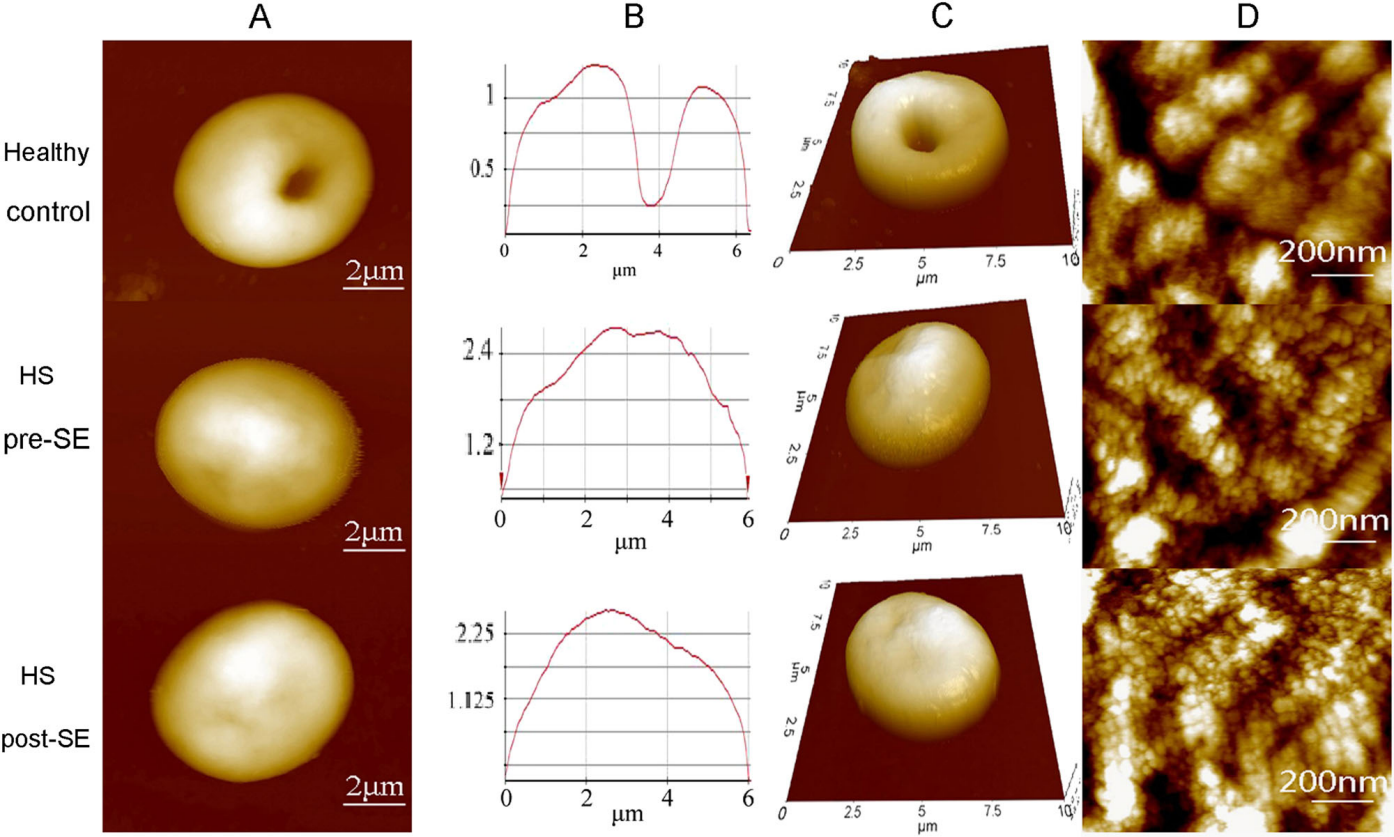
Representative AFM surface topographic images of erythrocytes from one healthy individual and one HS patient before and after splenectomy. **a** Single erythrocytes. **b** Height profile of the corresponding line in **a**. **c** 3D mode of single erythrocytes from **a**. **d** Surface ultrastructure on corresponding cells in images **a**. Scanning area: 10 μm × 10 μm in **a**, **b**, and **c**; 1 μm × 1 μm in **d**

In healthy individuals, normal erythrocytes were biconcave shaped (Figs. 2a and 2c), characterized by the well-organized nanoscale lattice corresponding to the underlying protein network. This network can be seen in high resolution AFM images (Fig. 2d).

**Fig. 3:**
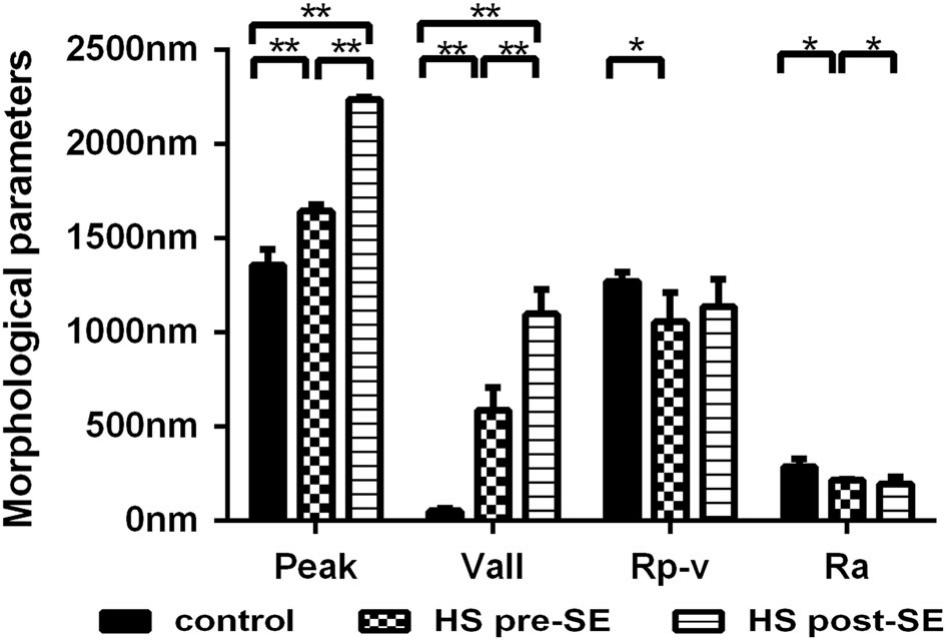
Comparison of erythrocyte morphological parameters. Peak: the maximum value of the z coordinate on the surface in the analyzed area; Vall: the minimum value of the z coordinate on the surface in the analyzed area; Rp-v: the difference between the peak and Vall; Ra: the average surface roughness of the erythrocyte. * *p* values < 0.05; ** *p* values < 0.01

Three months after the patients underwent the surgeries, the RBCs were examined again by AFM. The sizes of the cells increased, but they were still in spheroid shaped. In addition, the cells surface nanostructures were still abnormal, which indicated the defects of membrane proteins had no improvement (Fig. 3).

## Discussion

In this study, the nanostructure of abnormal RBCs from HS patients before and after splenectomy was studied with AFM. To validate the feasibility of using AFM to characterize RBCs, we first examined normal RBCs from healthy subjects and noted cells with a biconcave shape and regular cell surface lattice. This is consistent with the literature [26].

HS is characterized by a loss of membrane surface area leading to a reduced deformability due to defects in the membrane proteins ankyrin, band 3, β-spectrin, α-spectrin, and protein 4.2 [1]. Low magnification AFM images showed that the morphology of RBCs from HS patients were spheroid with no biconcave shape just like normal RBCs. The morphological AFM parameters were shown in Table 2 and the analysis showed the abnormal RBCs from HS patients had a significantly increased “Peak value” and significantly decreased “valley value” vs. normal RBCs. In addition, the abnormal RBCs had a significantly decreased Rp-v value vs. normal cells. This indicated that the membranes of RBCs from HS patients were flatter than that of the normal RBCs.

**Table 2:** The morphological AFM parameters of the three HS patients before and after splenectomy

	*L* (μm)	*W* (μm)	*H* (nm)	*h* (nm)	Rp-v(nm)	Ra(nm)
Pre-SE	5.92 ± 0.27**	5.81 ± 0.12**	1644.33 ± 34.93*	586.33 ± 120.50**	1058.00 ± 155.09*	215.67 ± 6.11*
Post-SE	6.12 ± 0.15*	5.85 ± 0.21*	2236.33 ± 15.63**	1098.67 ± 131.03**	1644.33 ± 34.93**	197.67 ± 36.20
Control	6.51 ± 0.27	6.22 ± 0.15	1358.33 ± 81.35	52.33 ± 15.04	1272.50 ± 48.79	287.00 ± 44.03

*L* length of the red blood cell, *W* width of the red blood cell, *H* maximum height of the cell, *h* the minimum height of the cell, Rp-v the valley to peak distance, *Ra* the average cell surface roughness, *Pre-SE* pre-splenectomy, *Post-SE* post-splenectomy

* *P* < 0.05 compared to the control group; ** *P* < 0.01 compared to the control group

The surface roughness (Ra) of the RBC plasma membrane is an important parameter reflecting the changes to the cell membrane skeleton [27]. We observed a significantly deceased Ra in the abnormal RBCs from HS patients vs. normal RBCs from healthy controls (215.67 ± 6.11 nm vs. 287.00 ± 44.03 nm, *P* = 0.025). Since Ra represented for the cell membrane skeleton, it indicated great differences from the RBC from HS patients and healthy individual since their Ra were statistically different, which led us to believe abnormalities in the cytoskeletal proteins in the HS cells [2, 3].

The RBCs were still spheroid in the HS patients even they underwent splenectomy. As shown in Table 2, despite the length of the RBCs increased vs. the pre-splenectomy samples, they were still smaller than normal RBCs. After splenectomy, the width of the RBCs only increased slightly. The RBCs from HS remained smaller than the normal RBCs. Furthermore, AFM topological analysis of the plasma membrane of RBCs indicated that the H and h of abnormal RBCs, which were 2236.33 ± 15.63 nm and 1098.67 ± 131.03 nm, respectively, were even higher than that measured pre-splenectomy (H = 1644.33 ± 34.93 nm, *P* < 0.001; and h = 586.33 ± 120.50 nm, *P* = 0.004). Our measurements about the pre-splenectomy, RBCs were consistent with the literature [28, 29]. The post-splenectomy measurements suggested that splenectomy had little effect on the defects of RBCs membrane proteins and cytoskeleton. Indeed, the abnormalities in the membrane ultrastructure were even more obvious.

Our AFM observations showed that although the size of RBCs increased after splenectomy, the abnormal RBCs were still spheroid. Further analysis of the plasma membrane ultrastructure indicated that splenectomy did not improve the defects in the protein and cytoskeleton of the RBC membrane. Furthermore, the size of the cell surface particles in abnormal RBCs was smaller than that of the normal RBCs. Our observations demonstrated that splenectomy does not improve the underlying genetic defects in the RBC plasma membrane. In fact, after splenectomy the underlying structural abnormalities in the RBCs of HS patients is even more obvious, as suggested by significantly decreased cells membrane roughness and smaller cell surface particles size (Fig. 2d).

## Conclusion

To the best of our knowledge, this is the first study investigating RBCs splenectomy changes in HS patients via AFM. In contrast to light microscopy that can only detect changes in RBC shapes and sizes, AFM offers structural details on the cell surface membranes, membrane proteins, and membrane nanostructures. While splenectomy markedly minimized the hemolytic anemia and other symptoms of HS, it didn’t reduce the abnormal cells in HS patients. And in some cases, it might even increase the number of abnormal cells because these cells loose a place to be destroyed. The underlying genetic defects can only be cured by gene therapy or allogeneic hematopoietic stem cell transplantation, while splenectomy can only relieve the symptoms of the patients. RBCs morphology we observed via AFM may provide additional information to aid HS diagnosis. Furthermore, AFM is a potential tool to investigate the pathophysiological mechanisms of HS and to monitor the treatment efficacy in HS patients. While AFM is limited by throughput, the relative SDs we observed here are generally <10 % and are consistent with that from flow cytometry. Future work will expand to higher cell counts to better understand the distribution in RBC morphology. Superior to flow cytometry and light microscopy, AFM can provide us more information the effects of novel treatment such as stem cell transplantation and it has higher value in the time of stem cell transplantation.
